# Are the Risk Factors for Bronchopulmonary Dysplasia and Retinopathy of Prematurity in Very Low-Birth-Weight Infants the Same?

**DOI:** 10.3390/children12040509

**Published:** 2025-04-15

**Authors:** Hui Wu, Juan Zhang, Jing Zhang, Yanhong Yu, Hua Zhang, Tongyan Han

**Affiliations:** Department of Pediatrics, Peking University Third Hospital, Beijing 100191, China; nemo107@bjmu.edu.cn (H.W.); 0763178448@bjmu.edu.cn (J.Z.); youkejingjing@bjmu.edu.cn (J.Z.); 2311210373@stu.pku.edu.cn (Y.Y.); zhanghua@bjmu.edu.cn (H.Z.)

**Keywords:** bronchopulmonary dysplasia, oxygen therapy, risk factors, retinopathy of prematurity, very low birth weight

## Abstract

**Background/Objectives**: Bronchopulmonary dysplasia (BPD) and retinopathy of prematurity (ROP) affect the prognosis of preterm infants, and their coexistence is a risk factor for poor long-term outcomes in very low-birth-weight infants. However, there has been limited in-depth assessment of common and independent risk factors for BPD and ROP within the same cohort. Therefore, we aimed to investigate the risk factors for BPD and ROP in very low-birth-weight infants born at ≤32 weeks of gestation and to explore the interaction between these two diseases. **Methods**: The participants were divided into four groups: BPD+ROP+, BPD-ROP-, BPD+ROP-, and BPD-ROP+. Gestational age, birth weight, maternal pregnancy complications, birth and postnatal diseases, and treatment conditions were compared among the groups. Subsequently, univariate and multivariate binary logistic regression analyses were conducted to explore the independent risk factors for BPD and ROP. **Results**: Common risk factors of BPD and ROP included gestational age and prolonged oxygen therapy. The multivariate analysis revealed that gestational age (OR: 4.44; 95% CI: 3–6.57), intubation resuscitation (OR: 2.35; 95% CI: 1.09–5.05), mechanical ventilation duration ≥ 7 days (OR: 1.01; 95% CI: 1.01–1.01), and prolonged total oxygen therapy (OR: 3.13; 95% CI: 1.28–7.64) were independent risk factors for BPD. Additionally, gestational age (OR: 0.66; 95% CI: 0.54–0.81) and prolonged oxygen therapy (OR: 1.02; 95% CI: 1–1.03) were independent risk factors for ROP. **Conclusions**: The proper control of the duration and concentration of oxygen therapy, along with the minimization of mechanical ventilation time, is crucial for reducing the incidence of both BPD and ROP.

## 1. Introduction

Bronchopulmonary dysplasia (BPD) and retinopathy of prematurity (ROP) impact the prognosis and quality of life of preterm infants, and their corresponding rates are increasing with progress in perinatal medicine. ROP incidence among preterm infants with a birth weight of <1500 g or gestational age (GA) < 30 weeks in the United States is 26.5% [1], and BPD incidence among preterm infants born at <32 weeks’ gestation is as high as 71.1% [2]. The coexistence of BPD and severe ROP is a high-risk factor for poor long-term outcomes in very low-birth-weight (VLBW) infants [3] and is associated with adverse neurodevelopmental outcomes in preterm infants without severe brain injury [4].

BPD is currently understood as a lung injury resulting from multiple prenatal, perinatal, and postnatal factors, predominately affecting immature lungs. ROP is a multifactorial disease characterized by incomplete retinal vascularization, leading to retinal ischemia and neovascularization and, in severe cases, retinal detachment and blindness [5]. Pathophysiological links have been established between BPD and ROP. Abnormal levels of angiogenesis regulators [6], immune-mediated inflammatory responses [7], and oxidative stress [8] play critical roles in the development of both diseases. Previous studies have explored the roles of GA, birth weight, the duration of oxygen therapy, and mechanical ventilation time in BPD and ROP [9,10,11]. Despite previous studies, few have systematically compared independent risk factors for BPD and ROP within the same cohort, which is crucial for understanding their shared and distinct pathophysiological mechanisms.

In this study, we aimed to analyze the clinical characteristics of BPD and ROP in a single-center cohort and explore their potential associations, common risk factors, and independent risk factors. We sought to provide a basis for clinical risk assessment and intervention strategies, further optimizing treatment strategies and outcome management for preterm infants.

## 2. Patients and Methods

### 2.1. Study Participants

#### 2.1.1. Source of Data

For this single-center, retrospective cohort study, we selected 579 VLBW infants with a GA of ≤32 weeks who were admitted to the neonatal intensive care unit (NICU) of Peking University Third Hospital between January 2016 and May 2022. This study was approved by the Ethics Committee of Peking University Third Hospital (approval number: [2019] Medical Ethics Review No. [231-01]), which waived the informed consent requirement.

#### 2.1.2. Inclusion Criteria

(1) Birth weight < 1500 g and GA ≤ 32 weeks.
(2) Born in the obstetrics department of our hospital and transferred to the NICU for treatment after birth.
(3) Survival to a postmenstrual age (PMA) of 36 weeks.

#### 2.1.3. Exclusion Criteria

(1) Severe congenital malformations, such as congenital lung diseases (e.g., congenital pulmonary hypoplasia, pulmonary sequestration, congenital bronchopulmonary cysts, transparent lungs, and congenital pulmonary arteriovenous fistulas), congenital heart diseases (e.g., pulmonary valve stenosis, aortic valve stenosis, and tetralogy of Fallot), or other systemic malformations.
(2) Incomplete medical history prior to PMA of 36 weeks due to factors such as death, discontinuation of treatment, self-discharge, or transfer to other hospitals for surgical treatment.

### 2.2. Diagnosis and Grouping

The diagnostic criteria for BPD were based on the 2018 revised standards [12]. For preterm infants with a GA of <32 weeks, BPD diagnosis requires imaging-confirmed persistent lung parenchymal abnormalities, with the need for continuous respiratory support for at least 3 consecutive days at a PMA of 36 weeks. The diagnosis of ROP was based on the international classification of ROP [13]. The first fundus screening was performed at 4 weeks post-birth or at a PMA of 32 weeks according to the Ministry of Health guidelines in China. The interval for subsequent screenings was determined based on the results of the previous examination. We performed bedside fundus ophthalmoscopy for the initial ROP screening. If further confirmation or monitoring of retinal changes was needed, RetCam photography was used for follow-up screening.

The study participants were grouped based on whether they met the diagnostic criteria for BPD and ROP. In the first part of the study, participants were divided into four groups: BPD+ROP+ (concurrent BPD and ROP), BPD+ROP- (BPD only), BPD-ROP+ (ROP only), and BPD-ROP- (neither BPD nor ROP). In the second part, all participants were further divided into BPD and non-BPD groups or ROP and non-ROP groups.

### 2.3. Data Collection and Definitions

The following data were collected through a review of medical records: (i) demographic information: sex, GA, birth weight, and whether the infant was small for GA (SGA); (ii) maternal information: prenatal corticosteroid use, presence of preeclampsia, and timing of premature rupture of membranes; (iii) birth information: 1 min Apgar score and whether intubation was required during neonatal resuscitation; (iv) postnatal diseases and treatment: these included neonatal respiratory distress syndrome (NRDS), hemodynamically significant patent ductus arteriosus (hsPDA), intracranial hemorrhage (grade ≥ 3), pulmonary hemorrhage, persistent pulmonary hypertension of the newborn (PPHN), intrauterine infectious pneumonia, early-onset sepsis, late-onset sepsis, hospital-acquired pneumonia, duration of mechanical ventilation, and total oxygen therapy duration.

The diagnosis of hsPDA requires at least one positive echocardiographic finding combined with three or more clinical symptoms [14]. Echocardiographic criteria include the following: (i) ductus arteriosus diameter ≥ 1.5 mm; (ii) ratio of left atrium to aortic root diameter ≥ 1.4; (iii) increased diastolic flow velocity in the left pulmonary artery; and (iv) aortic regurgitation. Clinical symptoms include the following: (i) systolic or continuous murmur heard along the left sternal border; (ii) increased precordial pulsation; (iii) Water-hammer pulse; (iv) heart rate > 180 beats/min at rest; (v) worsening respiratory status; and (vi) chest X-ray showing increased pulmonary vascular markings and/or cardiac enlargement, or presence of pulmonary edema.

Hospital-acquired pneumonia was diagnosed based on the presence of the following four items [15]: (i) onset of illness occurring > 48 h after admission; (ii) chest X-ray showing new, persistent, or progressive signs of pneumonia (e.g., consolidation or infiltration); (iii) clinical evidence of infection, with at least one of the following: unstable body temperature; apnea and/or bradycardia, tachypnea, or nasal flaring with inspiratory retraction; decreased (<5 × 10⁹/L) or markedly elevated (>20 × 10⁹/L) white blood cell count; neutrophil count <7.5 × 10⁹/L; elevated C-reactive protein level (>8 mg/L); and (iv) two or more of the following respiratory symptoms: deterioration of oxygenation and gas exchange, such as increased oxygen concentration or respiratory parameters on mechanical ventilation; new purulent sputum, changes in sputum characteristics, increased respiratory secretions, or more frequent suctioning.

Duration of mechanical ventilation refers to the total duration of endotracheal intubation from birth to the assessment point. If there were two or more instances of intubation, the total time for each intubation was summed and included in the analysis.

Total duration of oxygen therapy refers to the total time from birth to the assessment point during which oxygen concentration was >21%, including time spent on ambient oxygen, invasive, and noninvasive ventilation.

### 2.4. Statistical Analysis

Statistical analyses were conducted using R software (version 4.4.2, R Foundation for Statistical Computing, Vienna, Austria). Categorical variables are presented as frequencies and percentages, and continuous variables as mean ± standard deviation or median (interquartile range) for normally and non-normally distributed variables, respectively. Normality was assessed with the Shapiro–Wilk test.

Differences among the four groups were analyzed using the chi-square test for categorical variables and the Fisher exact test for small sample sizes. The Holm–Bonferroni method was applied to adjust *p*-values for multiple comparisons. For continuous variables, one-way ANOVA (with Tukey’s HSD post hoc test) was used for normally distributed data, and the Kruskal–Wallis H test (with Dunn’s post hoc test) for non-normally distributed data.

For comparisons between the two groups, independent *t*-tests and Mann–Whitney U tests were used for normally and non-normally distributed continuous variables, respectively. Multivariate binary logistic regression identified independent risk factors for BPD and ROP, with significance set at *p* < 0.05. Effect size was reported as odds ratios with 95% confidence intervals.

## 3. Results

### 3.1. Enrollment of Study Participants

**Figure 1:**
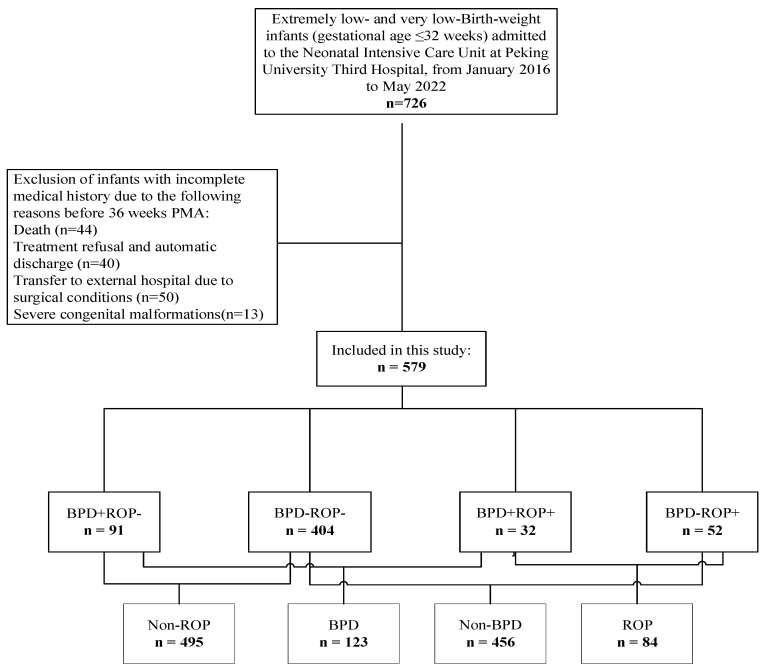
Flowchart of inclusion and grouping of study participants. BPD, bronchopulmonary dysplasia; PMA, postmenstrual age; ROP, retinopathy of prematurity.

The enrollment and grouping processes for the study participants are shown in Figure 1. The distribution and comparison of the variables across the four groups are presented in Table 1.

### 3.2. Pairwise Comparisons Among the Four Groups

#### 3.2.1. Comparison of Maternal Pregnancy and Birth Conditions

**Figure 2:**
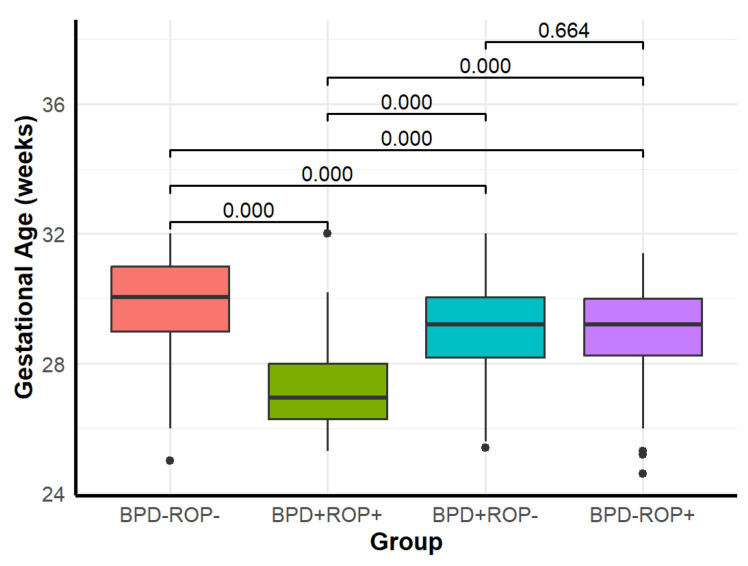
Distribution of gestational age in each group and pairwise comparisons.

**Figure 3:**
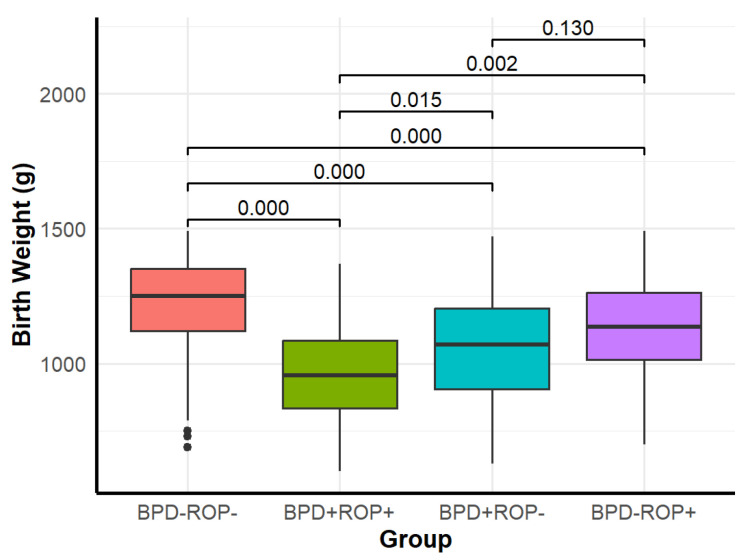
Distribution of birth weight in each group and pairwise comparisons.

**Figure 4:**
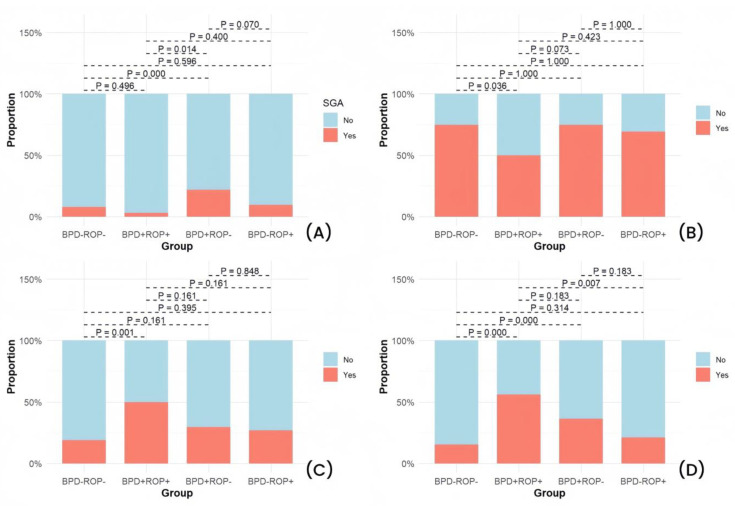
Pairwise comparisons of pregnancy and birth conditions: (**A**) percentage of small for GA (SGA) cases, (**B**) percentage of cases with adequate antenatal corticosteroids (ACS), (**C**) percentage of cases with 1 min Apgar score ≤ 7, and (**D**) percentage of cases with postnatal intubation during resuscitation.

Significant differences were observed among the four groups in GA, birth weight, SGA, adequate prenatal corticosteroid use, 1 min Apgar score ≤ 7, and the need for intubation during postnatal resuscitation (Table 1). Detailed results of the pairwise comparisons are shown in Figure 2, Figure 3 and Figure 4.

The pairwise comparison results showed that the BPD+ROP+ group had a significantly lower GA and birth weight than the other three groups. When compared with the BPD-ROP- group, both the BPD+ROP- and BPD-ROP+ groups exhibited lower GA and birth weight, with significant differences. The BPD+ROP+ group had more cases with 1 min Apgar score ≤ 7 and postnatal intubation compared to the BPD-ROP- group. The BPD+ROP- group had more cases requiring postnatal intubation compared to the BPD-ROP- group. Similarly, the BPD+ROP+ group had more cases requiring postnatal intubation compared to the BPD-ROP+ group.

#### 3.2.2. Comparison of Postnatal Diseases and Treatment Conditions

**Figure 5:**
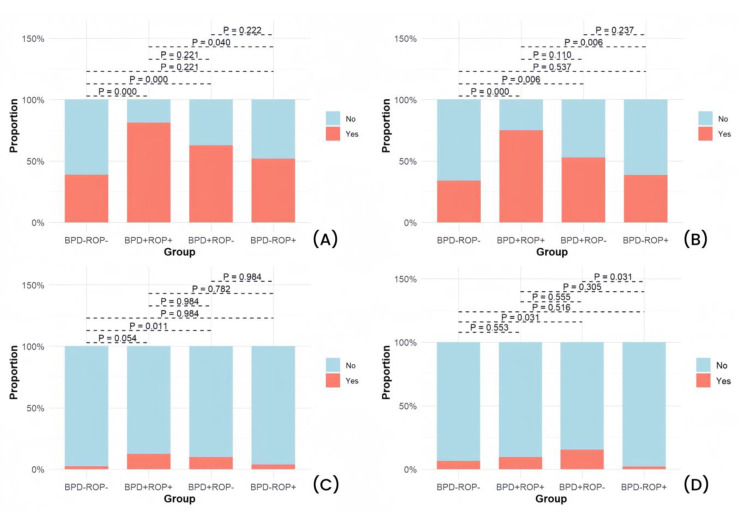
Pairwise comparisons of postnatal diseases: (**A**) percentage of neonatal respiratory distress syndrome cases, (**B**) percentage of hemodynamically significant patent ductus arteriosus cases, (**C**) percentage of pulmonary hemorrhage cases, and (**D**) percentage of persistent pulmonary hypertension of the newborn cases.

**Figure 6:**
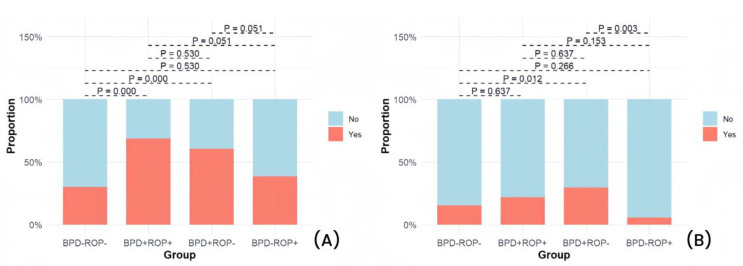
Pairwise comparisons of postnatal infectious diseases: (**A**) percentage of hospital-acquired pneumonia cases and (**B**) percentage of late-onset sepsis cases.

**Figure 7:**
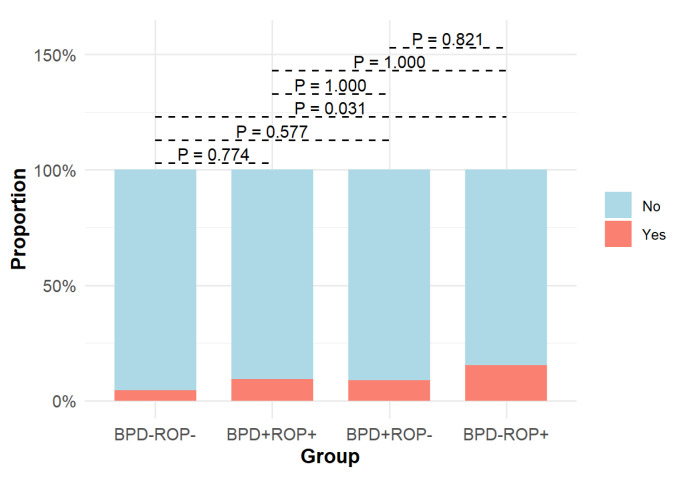
Percentage of cases with intracranial hemorrhage (grade ≥ 3) in the four groups and pairwise comparisons.

**Figure 8:**
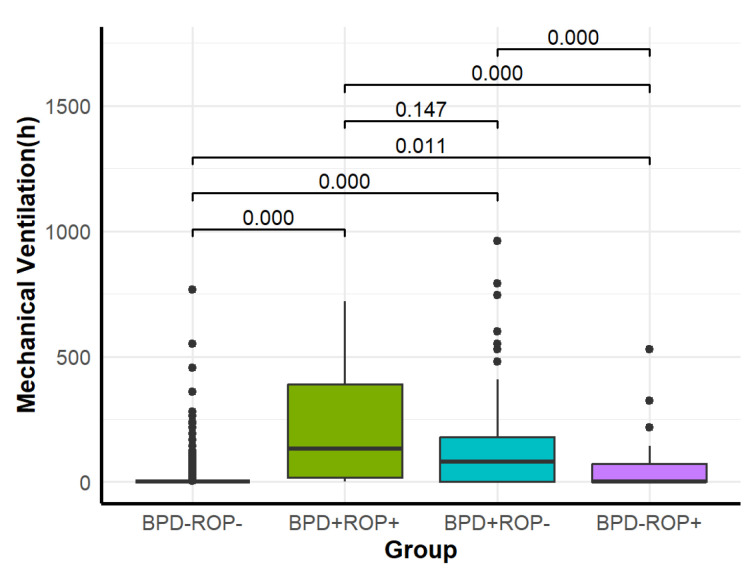
Comparison of mechanical ventilation duration among the four groups.

**Figure 9:**
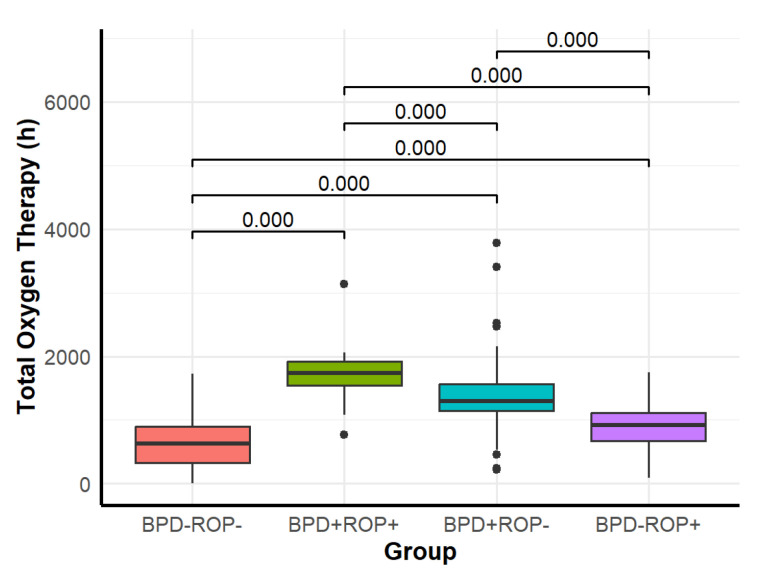
Comparison of total oxygen therapy duration among the four groups.

Among the four groups, significant differences were observed in NRDS, hsPDA, pulmonary hemorrhage, PPHN, intracranial hemorrhage (grade ≥ 3), hospital-acquired pneumonia, late-onset sepsis, mechanical ventilation duration, and total oxygen therapy duration (Table 1). Detailed results of pairwise comparisons are shown in Figure 5, Figure 6, Figure 7, Figure 8 and Figure 9.

The pairwise comparison results indicated that the BPD+ROP+ group had significantly higher incidences of NRDS and hsPDA than the BPD-ROP- and BPD-ROP+ groups. The BPD+ROP- group, when compared to the BPD-ROP- group, showed a significant increase in the incidence of NRDS and hsPDA. Additionally, the BPD+ROP- group had more cases of pulmonary hemorrhage compared to the BPD-ROP- group. The BPD+ROP- group had a significantly higher incidence of PPHN than the BPD-ROP- and BPD-ROP+ groups. The BPD-ROP+ group had significantly more cases of intracranial hemorrhage (grade ≥ 3) compared to the BPD-ROP- group. The incidence of hospital-acquired pneumonia was significantly higher in the BPD+ROP+ and BPD+ROP- groups than in the BPD-ROP- group. The BPD+ROP- group had a significantly higher incidence of late-onset sepsis than the BPD-ROP- and BPD-ROP+ groups.

Regarding mechanical ventilation, the BPD+ROP+, BPD+ROP-, and BPD-ROP+ groups had significantly longer durations than the BPD-ROP- group. The BPD+ROP+ and BPD+ROP- groups had significantly longer mechanical ventilation duration than the BPD-ROP+ group. Regarding total oxygen therapy, the BPD+ROP+ group had significantly longer duration compared to the other three groups, and the BPD+ROP- group had significantly longer oxygen therapy duration than the BPD-ROP- and BPD-ROP+ groups.

### 3.3. Independent Risk Factor Analysis for BPD and ROP

#### 3.3.1. Univariate Analysis of BPD Group vs. Non-BPD Group

The comparison between the BPD group and the non-BPD group showed significant differences in the following factors: sex, GA, birth weight, SGA, 1 min Apgar score ≤ 7, need for intubation during postnatal resuscitation, NRDS, hsPDA, PPHN, pulmonary hemorrhage, hospital-acquired pneumonia, late-onset sepsis, ROP, duration of mechanical ventilation, and total oxygen therapy duration (Table 2).

#### 3.3.2. Multivariate Analysis of BPD

The results revealed that GA, the need for intubation during postnatal resuscitation, mechanical ventilation duration ≥ 7 days, and prolonged total oxygen therapy duration are independent risk factors for the development of BPD in VLBW infants with GA ≤ 32 weeks (Table 3).

#### 3.3.3. Univariate Analysis of ROP Group vs. Non-ROP Group

The comparison between the ROP group and the non-ROP group showed significant differences in the following aspects: GA, birth weight, antenatal corticosteroid use, 1 min Apgar score ≤ 7, need for intubation postnatal resuscitation, NRDS, hsPDA, intracranial hemorrhage (grade ≥ 3), hospital-acquired pneumonia, BPD, mechanical ventilation duration, and total oxygen therapy duration (Table 4).

#### 3.3.4. Multivariate Analysis of ROP

The results revealed that a smaller GA and longer total oxygen therapy duration were independent risk factors for the occurrence of ROP in preterm infants with a GA ≤ 32 weeks and VLBW (Table 5).

## 4. Discussion

### 4.1. Common Risk Factors for BPD and ROP

This study included VLBW preterm infants with GA ≤ 32 weeks admitted to the NICU of a tertiary hospital in China from January 2016 to May 2022. The clinical data of the participants revealed that GA and total oxygen therapy duration played key roles in the occurrence of both BPD and ROP. Comparative analyses among the four groups, between the BPD and non-BPD groups, and between the ROP and non-ROP groups consistently highlighted GA as an independent risk factor in the occurrence of both BPD and ROP. The pathogenesis of BPD suggests that prematurity and low birth weight are the highest risk factors. In preterm infants, lung development may be abruptly interrupted during the canalicular or saccular stages, resulting in fundamental changes in lung morphology [16]. The incidence of BPD increases with decreasing GA and birth weight. Among preterm infants born at 22–24 weeks of gestation, approximately 80% are diagnosed with BPD, and 95% of these infants with BPD have VLBW [12]. Previous research has shown that GA and birth weight are independent risk factors for ROP [10], and the incidence of ROP increases with decreasing GA and birth weight [16]. This aligns with the pathophysiology and mechanisms of ROP. Retinal vascular development in the fetus begins at 16 weeks of gestation from the optic disk and progresses to the nasal retina by 32 weeks, with full vascularization occurring in the temporal retina by 36–40 weeks. Therefore, lower GA results in less mature retinal vascular development. Preterm infants continue to experience retinal oxygen deprivation and abnormal proliferation of retinal vessels owing to various high-risk factors, ultimately leading to the development of ROP [5].

In the present study, the GA and birth weight in the BPD+ROP group, as well as in the BPD-only and ROP-only groups, were significantly lower than those in the control (BPD-ROP-) group. However, the multivariate logistic regression analysis did not indicate birth weight as an independent risk factor for ROP or BPD. This may be attributed to the relatively homogeneous study population (i.e., preterm infants ≤ 32 weeks GA) and the high correlation between GA and birth weight. Smaller GA is often associated with lower birth weight, which explains why the independent effect of birth weight was captured by GA in multivariate modeling.

Prolonged oxygen therapy is another common risk factor for both BPD and ROP. For example, Wickramasinghe et al. [8] exposed neonatal mice to a 75% oxygen environment for 12 days and observed pathological changes in their eyes and lungs. They found vascular occlusion and new blood vessels in the eyes, similar to those observed in ROP, airway developmental abnormalities, and simplified alveoli resembling BPD pathology. Similar findings have been reported in clinical studies. Carlo and Higgins [17] showed that the duration and concentration of oxygen therapy are significantly associated with the risk of ROP. Long-term exposure to high oxygen concentrations, especially when the duration of oxygen therapy is prolonged, significantly increases the risk of developing ROP. However, the relationship between BPD and the duration of oxygen therapy is more complex; it is not simply a cause-and-effect relationship. Prolonged oxygen therapy may either trigger or result from BPD.

### 4.2. Differences in Risk Factors for BPD and ROP

In the four-group comparison, we observed significant differences in many clinical characteristics between infants with both BPD and ROP, those with only BPD, and the other two groups, particularly in GA, birth weight, resuscitation interventions, and the duration of mechanical ventilation.

Infants with both BPD and ROP, as well as those with only BPD, had higher rates of intubation during resuscitation and significantly longer mechanical ventilation times than the ROP-only and control (BPD-ROP-) groups. This finding suggests that intubation during resuscitation and prolonged mechanical ventilation play critical roles in the development of BPD, particularly in VLBW infants. The multivariate analysis further confirmed that mechanical ventilation time ≥ 7 days is an independent risk factor for BPD, which is consistent with the higher mechanical ventilation demand observed in the BPD group (whether isolated or co-occurring with ROP). Berger et al. [18] found that in 262 preterm infants with GA ≤ 28 weeks, the risk of developing BPD increased when extubation was delayed beyond the first week of life. Jensen et al. [19] conducted a retrospective cohort study on extremely low-birth-weight infants and found that the risk of developing BPD increased with longer mechanical ventilation times. While our study focused on the duration of mechanical ventilation, it is important to consider that the frequency and severity of intubation events may also play a role in the pathogenesis of BPD. Glenn et al. [20] highlighted that increased intubation attempts in the first week of life are associated with a higher risk of BPD, suggesting that repeated intubations may contribute to mechanical lung injury and subsequent BPD development. In their study, each additional intubation attempt increased the odds of developing BPD (OR = 1.29; CI 1.03–1.62; *p* = 0.029). Future studies should explore the relationship between intubation frequency and BPD severity, incorporating detailed data on intubation attempts to clarify the role of repeated intubations in lung injury and BPD progression.

Infants with only ROP exhibited a lower demand for mechanical ventilation and intubation. The multivariate analysis revealed that mechanical ventilation time was not an independent risk factor for ROP. Previous studies have debated the role of mechanical ventilation in ROP development. Protsyk and García Serrano [21] found that each additional day of mechanical ventilation increased the risk of ROP treatment by 1.05 times, with a positive correlation between the duration of mechanical ventilation and the risk of ROP. Infants with longer mechanical ventilation times had a significantly higher likelihood of developing ROP requiring treatment. However, Li et al. [22] found that prolonged noninvasive ventilation were the primary risk factors for ROP in preterm infants with BPD. In this study, differences in mechanical ventilation time were not observed, likely owing to the inclusion of total oxygen therapy time, which comprises mechanical ventilation, noninvasive ventilation, and normobaric oxygen therapy. These findings suggest that the duration of oxygen exposure may play a more significant role in ROP development compared to the mechanical injury caused by ventilation.

The four-group comparison results revealed that the BPD-only group had a higher proportion of cases with hospital-acquired pneumonia and late-onset sepsis than the ROP-only group. Kim et al. [23] found a link between late-onset sepsis and the development of moderate-to-severe BPD, suggesting that inflammatory mediators and cells entering the lungs via the bloodstream increase the risk of BPD. Sucasas et al. [24] identified hospital-acquired infections as an independent risk factor for BPD, which correlated with both increased risk and greater severity of BPD. Several studies have investigated the association between inflammation and ROP. Glaser et al. [25] analyzed data from 12,794 extremely preterm infants in the German Neonatal Network and found that sepsis was significantly associated with the risk of ROP and the need for ROP treatment. Dammann et al. [26] revealed that maternal inflammation during pregnancy may play a role in the early stages of ROP. In the present study, although the univariate analysis indicated a significantly higher proportion of hospital-acquired pneumonia in the ROP group than in the non-ROP group, the multivariate analysis did not identify it as an independent risk factor. The higher incidence of hospital-acquired infections in the BPD-only group than in the ROP-only group suggests that inflammation may play a more significant role in the development of BPD.

Infants with only ROP showed a higher proportion of severe intraventricular hemorrhage (≥3rd degree) than those in the control (BPD-ROP-) group. Additionally, the univariate analysis for ROP showed a significant difference in severe intraventricular hemorrhage incidence compared to that in the non-ROP group. Jung and Moon [27] found that intraventricular hemorrhage was a risk factor for ROP, and Chang et al. [28] showed that intraventricular hemorrhage was associated with ROP progression. The exact mechanism linking intraventricular hemorrhage and ROP remains unclear; however, some studies suggest that both intraventricular hemorrhage and ROP share similar characteristics with pathological changes related to immature blood vessels and an unstable oxygen supply [29].

### 4.3. Interaction Between BPD and ROP

In the four-group comparison, infants with both BPD and ROP had a lower GA and birth weight and required significantly longer oxygen therapy than infants with only BPD or ROP. This finding suggests that the occurrences of BPD and ROP are not independent. Moreover, univariate analysis for both BPD and ROP indicated that the proportion of infants with ROP was significantly higher in the BPD group than in the non-BPD group. Conversely, the proportion of infants with BPD was significantly higher in the ROP group than in the non-ROP group.

Previous studies have reported similar findings. Li et al. [22] identified a positive correlation between BPD severity and the incidence and severity of ROP. Podraza et al. [9] found that preterm infants with BPD had more severe ROP than those without BPD. These findings suggest that, although BPD and ROP have distinct independent risk factors, the interaction between the two and some shared underlying pathogenic mechanisms may exacerbate the risk of both diseases.

This study has certain limitations. First, it was conducted at a single tertiary NICU with a relatively small sample size, which may limit the generalizability of the results. Future studies should consider expanding the sample size and conducting multicenter studies to enhance the external validity of our findings. Second, as a retrospective study, data were obtained from electronic medical records. Infants with incomplete medical records, due to factors such as death, treatment refusal, self-discharge, transfer to other hospitals, or severe congenital malformations, were excluded during the enrollment process. As this is a retrospective study, we were unable to further analyze the characteristics of the excluded infants, which may have introduced potential bias. Furthermore, this study mainly focused on early clinical data without long-term follow-up. Future prospective cohort studies with longer follow-up periods are required to investigate the long-term effects of BPD and ROP on neurodevelopmental and visual outcomes. Third, although a multivariate analysis was performed, some confounding factors were not included in the statistical analysis (e.g., the number of blood transfusions and maternal comorbidities such as diabetes). Previous studies demonstrated that these factors may influence the occurrence of BPD and ROP. Additionally, this study did not explore inflammatory markers (e.g., C-reactive protein and interleukins), which play a central role in both BPD and ROP. The omission of these data represents a missed opportunity to better understand the inflammatory mechanisms underlying these conditions. Moreover, specific details regarding oxygen therapy management, such as oxygen concentration and ventilation methods, were not further refined or stratified, which may have affected the results. Future studies should aim to collect more comprehensive data on the study population and consider the combined effects of oxygen concentration and ventilation methods. Furthermore, owing to the limited sample size, this study did not investigate the severity of BPD or ROP. Future studies should further analyze the risk factors and interactions of different severity levels of BPD and ROP.

## 5. Conclusions

Although BPD and ROP share overlapping risk factors, such as GA and the duration of oxygen therapy, their independent risk factors demonstrate different clinical characteristics. BPD is influenced by mechanical ventilation and intubation resuscitation, whereas ROP is more closely related to the duration of oxygen therapy. This suggests that prevention and management strategies for BPD and ROP should be tailored to their specific risk profiles despite their shared pathological backgrounds. Although no new risk factors were identified, our study still provides valuable support for the current knowledge. Based on our study findings and the supporting literature, we recommend a more cautious approach to oxygen therapy management in preterm infants, particularly in high-risk infants with extremely low birth weights and extremely premature GA. Optimizing and individualizing oxygen therapy protocols, shortening the duration of oxygen therapy, and minimizing mechanical ventilation may reduce the risk of BPD and ROP, thereby improving the prognosis of preterm infants.

## Figures and Tables

**Table 1:** Comparison of baseline characteristics.

Variable	BPD-ROP- (n = 404)	BPD+ROP+ (n = 32)	BPD+ROP- (n = 91)	BPD-ROP+ (n = 52)	χ^2^/Z	*p*-Value
Male sex	203 (50.25%)	20 (62.5%)	56 (61.54%)	22 (42.31%)	7.185	0.066
Gestational age (weeks)	30.05 (29, 31)	26.95 (26.3, 28)	29.2 (28.2, 30.05)	29.2 (28.25, 30)	72.823	<0.001
Birth weight (g)	1250 (1120, 1352.5)	955 (835, 1085)	1070 (905, 1205)	1135 (1015, 1262.5)	88.722	<0.001
SGA	32 (7.92%)	1 (3.12%)	20 (21.98%)	5 (9.62%)	18.108	<0.001
Antenatal corticosteroids	302 (74.75%)	16 (50%)	68 (74.73%)	36 (69.23%)	9.7	0.021
Maternal preeclampsia	151 (37.38%)	10 (31.25%)	29 (31.87%)	16 (30.77%)	1.903	0.593
Premature rupture of membranes > 18 h	86 (21.29%)	5 (15.62%)	23 (25.27%)	11 (21.15%)	1.43	0.698
1 min Apgar score ≤ 7	77 (19.06%)	16 (50%)	27 (29.67%)	14 (26.92%)	19.362	<0.001
Endotracheal intubation during resuscitation	62 (15.35%)	18 (56.25%)	33 (36.26%)	11 (21.15%)	43.837	<0.001
NRDS	157 (38.86%)	26 (81.25%)	57 (62.64%)	27 (51.92%)	35.155	<0.001
hsPDA	137 (33.9%)	24 (75%)	48 (52.7%)	20 (38.5%)	28.849	<0.001
PPHN	26 (6.44%)	3 (9.38%)	14 (15.38%)	1 (1.92%)	11.164	0.011
Pulmonary hemorrhage	9 (2.23%)	4 (12.5%)	9 (9.89%)	2 (3.85%)	16.931	0.001
Intraventricular hemorrhage (grade ≥ 3)	18 (4.46%)	3 (9.38%)	8 (8.79%)	8 (15.38%)	10.914	0.012
Early-onset sepsis	8 (1.98%)	1 (3.12%)	1 (1.1%)	3 (5.77%)	-	0.218
Intrauterine infectious pneumonia	68 (16.83%)	6 (18.75%)	20 (21.98%)	9 (17.31%)	1.374	0.712
Hospital-acquired pneumonia	122 (30.2%)	22 (68.75%)	55 (60.44%)	20 (38.46%)	42.804	<0.001
Late-onset sepsis	62 (15.35%)	7 (21.88%)	27 (29.67%)	3 (5.77%)	16.245	0.001
Mechanical ventilation time (h)	0 (0, 0)	132 (18.5, 390)	80 (0, 180)	0 (0, 72)	120.498	<0.001
Total oxygen therapy time (h)	624 (323, 897.75)	1740 (1548, 1920)	1296 (1140, 1560)	924 (669.25, 1110)	220.831	<0.001

BPD, bronchopulmonary dysplasia; ROP, retinopathy of prematurity; SGA, small for gestational age; NRDS, neonatal respiratory distress syndrome; hsPDA, hemodynamically significant patent ductus arteriosus; PPHN, persistent pulmonary hypertension.

**Table 2:** Univariate analysis of BPD and non-BPD groups.

Variable	Non-BPD (n = 456)	BPD (n = 123)	χ^2^/Z	*p*-Value
Male sex	231 (50.66%)	47 (38.21%)	6.012	0.014
Gestational age (weeks)	30 (28.6, 31)	28.6 (27.35, 30)	6.391	<0.001
Birth weight (g)	1240 (1100, 1350)	1050 (875, 1175)	8.396	<0.001
SGA	37 (8.11%)	21 (17.07%)	8.626	0.003
Antenatal corticosteroids	338 (74.12%)	84 (68.29%)	1.666	0.197
Maternal preeclampsia	167 (36.62%)	39 (31.71%)	1.021	0.312
Premature rupture of membranes > 18 h	97 (21.27%)	28 (22.76%)	0.127	0.721
1 min Apgar score ≤ 7	91 (19.96%)	43 (34.96%)	12.259	<0.001
Endotracheal intubation during resuscitation	73 (16.01%)	51 (41.46%)	37.295	<0.001
NRDS	37.295	<0.001	28.691	<0.001
hsPDA	157 (34.43%)	72 (58.54%)	23.546	<0.001
PPHN	27 (5.92%)	17 (13.82%)	8.610	0.003
Pulmonary hemorrhage	11 (2.41%)	13 (10.57%)	16.221	<0.001
Intraventricular hemorrhage (grade ≥ 3)	26 (5.7%)	11 (8.94%)	1.701	0.192
Early-onset sepsis	11 (2.41%)	2 (1.63%)	0.032	0.858
Intrauterine infectious pneumonia	77 (16.89%)	26 (21.14%)	1.198	0.274
Hospital-acquired pneumonia	142 (31.14%)	77 (62.6%)	40.771	<0.001
Late-onset sepsis	65 (14.25%)	34 (27.64%)	12.249	<0.001
Mechanical ventilation time (h)	0 (0, 19.25)	96 (0, 216)	−10.698	<0.001
Total oxygen therapy time (h)	672 (336, 936)	1392 (1200, 1728)	−14.241	<0.001
ROP	52 (11.4%)	32 (26.02%)	16.677	<0.001

BPD, bronchopulmonary dysplasia; SGA, small for gestational age; NRDS, neonatal respiratory distress syndrome; hsPDA, hemodynamically significant patent ductus arteriosus; PPHN, persistent pulmonary hypertension of the newborn; ROP, retinopathy of prematurity.

**Table 3:** Multivariate analysis of BPD.

Variable	β	SE	Wald	*p*-Value	Odds Ratio	95% Confidence Interval
Constant	−56.710	7.003	−8.098	<0.001		
Gestational age (weeks)	1.490	0.200	7.438	<0.001	4.44	3–6.57
Endotracheal intubation during resuscitation	0.854	0.391	2.184	0.029	2.35	1.09–5.05
Mechanical ventilation time ≥7 days	0.010	0.001	9.279	<0.001	1.01	1.01–1.01
Total oxygen therapy time (h)	1.141	0.455	2.509	0.012	3.13	1.28–7.64

**Table 4:** Univariate analysis of ROP and non-ROP groups.

Variable	Non-ROP (n = 495)	ROP (n = 84)	χ^2^/Z	*p*-Value
Male sex	236 (47.68%)	42 (50%)	0.155	0.694
Gestational age (weeks)	29.6 (28.6, 30.6)	28.4 (26.58, 29.4)	6.506	<0.001
Birth weight (g)	1220 (1080, 1350)	1080 (877.5, 1210)	5.605	<0.001
SGA	52 (10.51%)	6 (7.14%)	0.901	0.343
Antenatal corticosteroids	370 (74.75%)	52 (61.9%)	5.993	0.014
Maternal preeclampsia	180 (36.36%)	26 (30.95%)	0.917	0.338
Premature rupture of membranes > 18 h	109 (22.02%)	16 (19.05%)	0.375	0.54
1 min Apgar score ≤ 7	104 (21.01%)	30 (35.71%)	8.729	0.003
Endotracheal intubation during resuscitation	95 (19.19%)	29 (34.52%)	10.030	0.002
NRDS	214 (43.23%)	53 (63.1%)	11.402	0.001
hsPDA	185 (37.37%)	44 (52.38%)	6.765	0.009
PPHN	40 (8.08%)	4 (4.76%)	1.127	0.289
Pulmonary hemorrhage	18 (3.64%)	6 (7.14%)	1.427	0.232
Intraventricular hemorrhage (grade ≥ 3)	26 (5.25%)	11 (13.1%)	7.384	0.007
Early-onset sepsis	9 (1.82%)	4 (4.76%)	1.653	0.199
Intrauterine infectious pneumonia	88 (17.78%)	15 (17.86%)	0.000	0.986
Hospital-acquired pneumonia	177 (35.76%)	42 (50%)	6.194	0.013
Late-onset sepsis	89 (17.98%)	10 (11.9%)	1.870	0.172
Mechanical ventilation time (h)	0 (0, 48)	22 (0, 126)	−4.209	<0.001
Total oxygen therapy time (h)	724 (407, 1102)	1152 (851.25, 1656)	−6.532	<0.001
BPD	91 (18.38%)	32 (38.1%)	16.677	<0.001

ROP, retinopathy of prematurity; SGA, small for gestational age; NRDS, neonatal respiratory distress syndrome; hsPDA, hemodynamically significant patent ductus arteriosus; PPHN, persistent pulmonary hypertension of the newborn; BPD, bronchopulmonary dysplasia.

**Table 5:** Multivariate analysis of ROP.

Variable	β	SE	Wald	*p*-Value	Odds Ratio	95% Confidence Interval
Constant	9.476	3.154	3.004	0.003		
GA (weeks)	−0.412	0.103	−3.994	<0.001	0.66	0.54–0.81
Total oxygen therapy time (h)	0.017	0.007	2.383	0.017	1.02	1–1.03

ROP: retinopathy of prematurity, GA: gestational age.

## Data Availability

The data presented in this study are only available on request from the corresponding author due to the author’s workplace policy on medical data.

## Abbreviations

The following abbreviations are used in this manuscript:

BPD	bronchopulmonary dysplasia
GA	gestational age
hsPDA	hemodynamically significant patent ductus arteriosus
NICU	neonatal intensive care unit
NRDS	neonatal respiratory distress syndrome
PMA	postmenstrual age
PPHN	persistent pulmonary hypertension of the newborn
ROP	retinopathy of prematurity
SGA	small for gestational age
VLBW	very low birth weight
